# Analysis and correction of meteorological disturbance observed by ground radars in complex environment

**DOI:** 10.1371/journal.pone.0258168

**Published:** 2021-10-04

**Authors:** Jijun Wang, Xiao Zhou, Songlin Yu, Bingzhen Li, Yan Li

**Affiliations:** Institute of National Defense Engineering, Academy of Military Sciences, Beijing, China; Universidade Federal de Uberlandia, BRAZIL

## Abstract

Ground radar interferometry technology, as a new tool for active remote sensing, has been widely used in the detection of a variety of targets, including landslides, bridges, mines, and dams. This technique usually employs a continuous observation mode with no space baseline. The detection accuracy is mainly affected by meteorological disturbances and noise in the observation environment. In a complex observation environment, meteorological disturbances can lead to phase errors of 10 mm or more, and the effects are different in the range and azimuth directions; this can seriously affect the accuracy of the measurement. In this paper, we analyze the spatial distribution of the phase of meteorological disturbances based on radar monitoring experiments in a complex environment, and propose a correction method that reduces the atmospheric disturbance phase to less than 0.6 mm and effectively improves radar observation accuracy.

## Introduction

Ground-based radar interferometry technology possesses such advantages as a short sampling period and high spatial resolution, and can detect the sub-millimeter level of deformation of a target. It can be used to effectively monitor the deformation of crucial targets like dams and landslides in a small region. It has gradually become the focus of earthquake mitigation across the globe [1, 2]. This technology has freed space-borne and airborne radars from the limitations of long sampling period and low spatial resolution, and has greatly improved the application range of radar interferometry technology by constructing the proper geometric imaging relationship according to monitoring requirements [3, 4]. Researchers have conducted extensive experiments on the monitoring of target deformation, such as that of landslides [5], dams [6], bridges [7], and glaciers [8], and verified the feasibility and advantages of the technology in many fields.

Ground-based radar interferometry technology does not need a spatial baseline, and its monitoring accuracy is mainly affected by meteorological disturbances and noise in the observation environment. All existing atmospheric correction methods are based on the assumption of atmospheric homogeneity. Different correction methods are proposed for different applications by establishing a geometric relationship between the distance from the radar to the target and the influence of meteorological disturbances [9–11]. In a complex observation environment, however, such as an environment that contains a large amount of water vapor, it is often difficult to effectively suppress the influence of meteorological disturbances on phase interference and hence on the measurement accuracy of the technique. Therefore, a meteorological disturbance correction method that takes into account the spatial position information of the target and corrects the results based on the characteristics of the target region is proposed. Through comparison with existing methods, the effectiveness of the proposed method is verified.

In Section 1 of this paper, we first analyze the commonly used meteorological correction models, and then in Section 2 we analyze the distribution of meteorological disturbances in the range and azimuth directions for a complex observation scene and propose a meteorological disturbance correction method based on spatial distribution. In Section 3, we perform statistical analysis of the atmospheric disturbance phases of landmark buildings such as power plants and dams in the observation scene, and compare the corrected results using different methods. Finally, we summarize the meteorological disturbance phase distribution characteristics, correction methods, and experimental results.

## Meteorological correction method

The signal propagation delay caused by atmospheric influences (humidity and temperature fluctuations) decreases as propagation wavelength increases. For a ground-based radar signal traveling over a short distance, it may be regarded as propagating along a straight path [12]:

$$d_{atm,p(i)}=10^{-6}\int_{L_{P}}N(r(\vec{l}),i)dl$$ (1)

Here, *L*_*p*_ represents the delayed path of the target and *N* the atmospheric delay index, which is related to the spatial position $r(\vec{l})$ of the target and the signal acquisition time *i*. When the frequency of the radar wave is *f*_*c*_ and the distance between the target point and the radar is *r*_*n*_, it is assumed that the atmospheric delay of a stable point in the observation zone is affected only by time *I*; that is, its variation is independent of distance *r*_*n*_ and the atmosphere is stable within the propagation time between radar and the target, and then the acquired phase value of the radar *φ*(*i*) is [13]

$$\varphi (i)=\frac{4\pi f_{c}r_{n}n(i)}{c}$$ (2)

where *c* is the speed of light, *n*(*i*) the atmospheric refraction coefficient at time *i*, and its relationship with *N* in Eq (1) is expressed as follows:

$$N=(n-1)\times 10^{6}$$ (3)

Therefore, the phase difference Δ*φ* of the stable point due to atmospheric changes during different observation periods is

$$\Delta \varphi =\frac{4\pi f_{c}r_{n}}{c}(n(i_{2})-n(i_{1}))=\frac{4\pi f_{c}r_{n}}{c}•\Delta n$$ (4)

When performing atmospheric corrections, under the premise of atmospheric homogeneity, a linear relationship exists between the phase difference caused by meteorological disturbances in the observation scenario and the distance between the target and the radar. Taking into account the effects of noise and other high-order influencing factors, a second-order polynomial such as that shown in Eq (5) is constructed to fit the meteorological disturbance characteristics in the observation scenario. Using the meteorological disturbance value obtained at the stable point and the distance from the point to the radar, the coefficients in the equation are solved for, and then corrected for the meteorological effect for target points in different positions:

$$\varphi_{atm}=a•r^{2}+b•r$$ (5)

where *r* is the distance between the target point and the radar, *φ*_*atm*_ the influence value of the meteorological disturbance, and *a* and *b* coefficients of the equation.

## Meteorological disturbance analysis in complex environment

**Fig 1:**
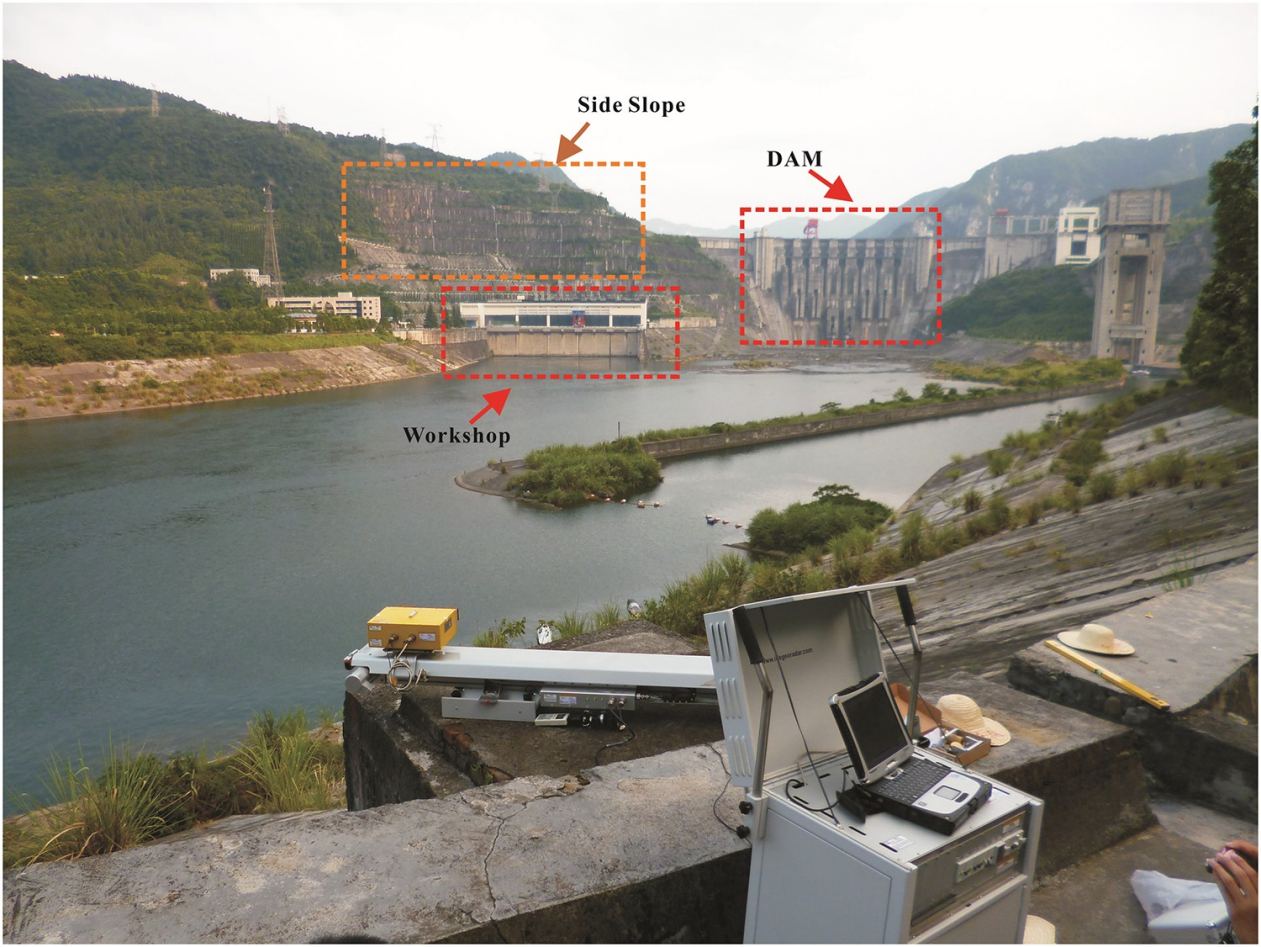
Monitoring site map.

**Fig 2:**
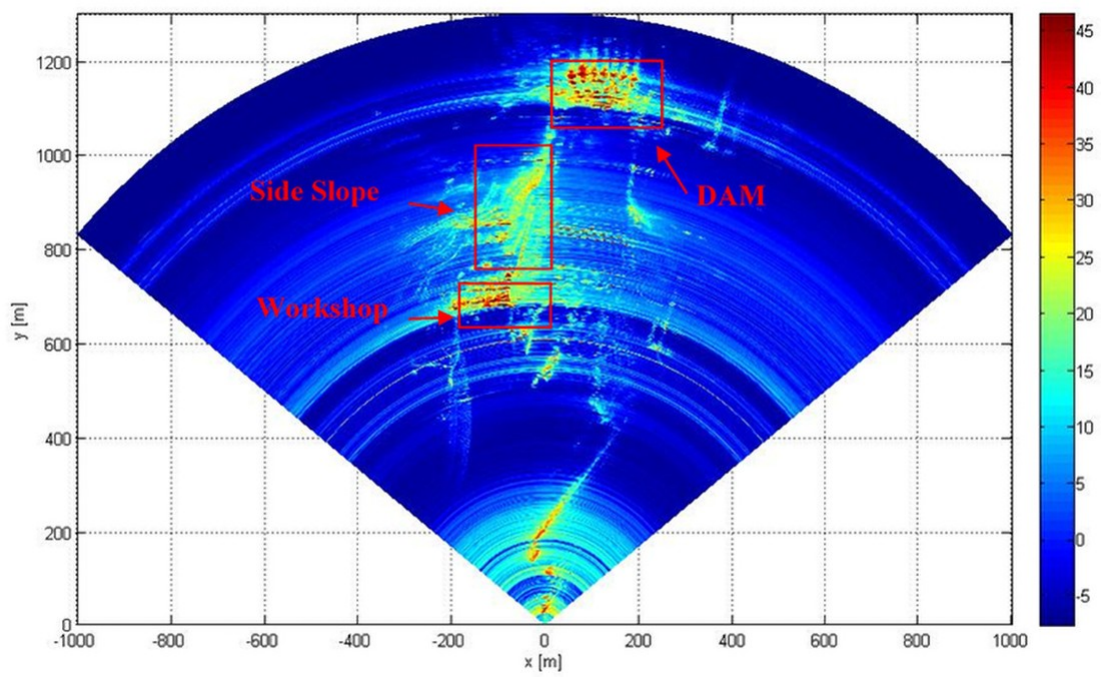
Radar signal intensity diagram.

Under normal circumstances, the changes of the meteorological factors are the most violent around noon. In a continuous monitoring experiment conducted on a ground radar across the river on the main dam of the Yanshui Power Station in Yichang, Hubei Province, China, and on the slope and power-plant buildings on the right-hand bank from 11:07 to 14:03, a total of 31 radar images were acquired. Figs 1 and 2 show the onsite surveillance map and radar signal intensity map, respectively. Fig 1 shows that there is little vegetation on the main dam structure, side slope, and around the power plant. The main dam and side slope are constructed out of concrete and there are metal buildings in the plant area. Fig 2 shows that in the observation zone the radar echoes from the main dam, side slope, and plant buildings are quite strong and that extensive resolution elements can be acquired for meteorological analysis. Radio echoes from other targets are weak due to angle and signal attenuation, but scattered radar echoes are still strong enough to serve as resolution elements for analysis. Targets located close to the radar provided strong echoes due to the short propagation distance and low attenuation of the signal.

**Fig 3:**
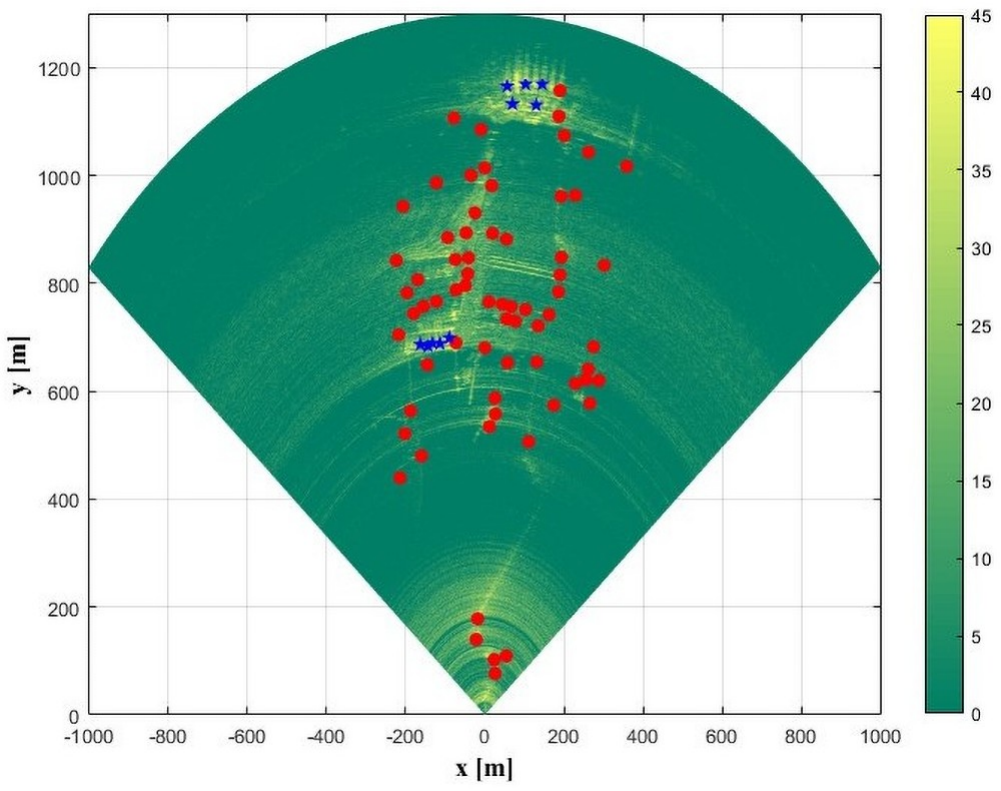
Distribution of selected points.

Owing to the short observation time, it is assumed that the targets experience no physical deformation during the observation and that the target interferometric phase is mainly affected by atmospheric disturbances and noise. Taking advantage of the strong scattering intensity, high stability, and immunity to noise characteristics of permanent scatterer (PS) points, a choice was made to perform meteorological disturbance analysis on PS points in the observation zone. The PS points were chosen based on multiple thresholds, including amplitude threshold, correlation coefficient threshold, and amplitude discreteness index. In this work, the amplitude threshold was set to 35 dB, the correlation coefficient to 0.9, and the amplitude discreteness index to 0.15. Combined with the distribution characteristics of PS points in different regions, 66 PS points were finally uniformly chosen, and the distribution of their positions is shown in Fig 3 as circular points.

From Eq (4), the atmospheric refractive index *n*(*i*) at different observation periods is given by

$$n(i)=n(1)+\Delta n(i)=\frac{c•\Delta \varphi (i)}{4\pi f_{c}r_{n}}$$ (6)

**Fig 4:**
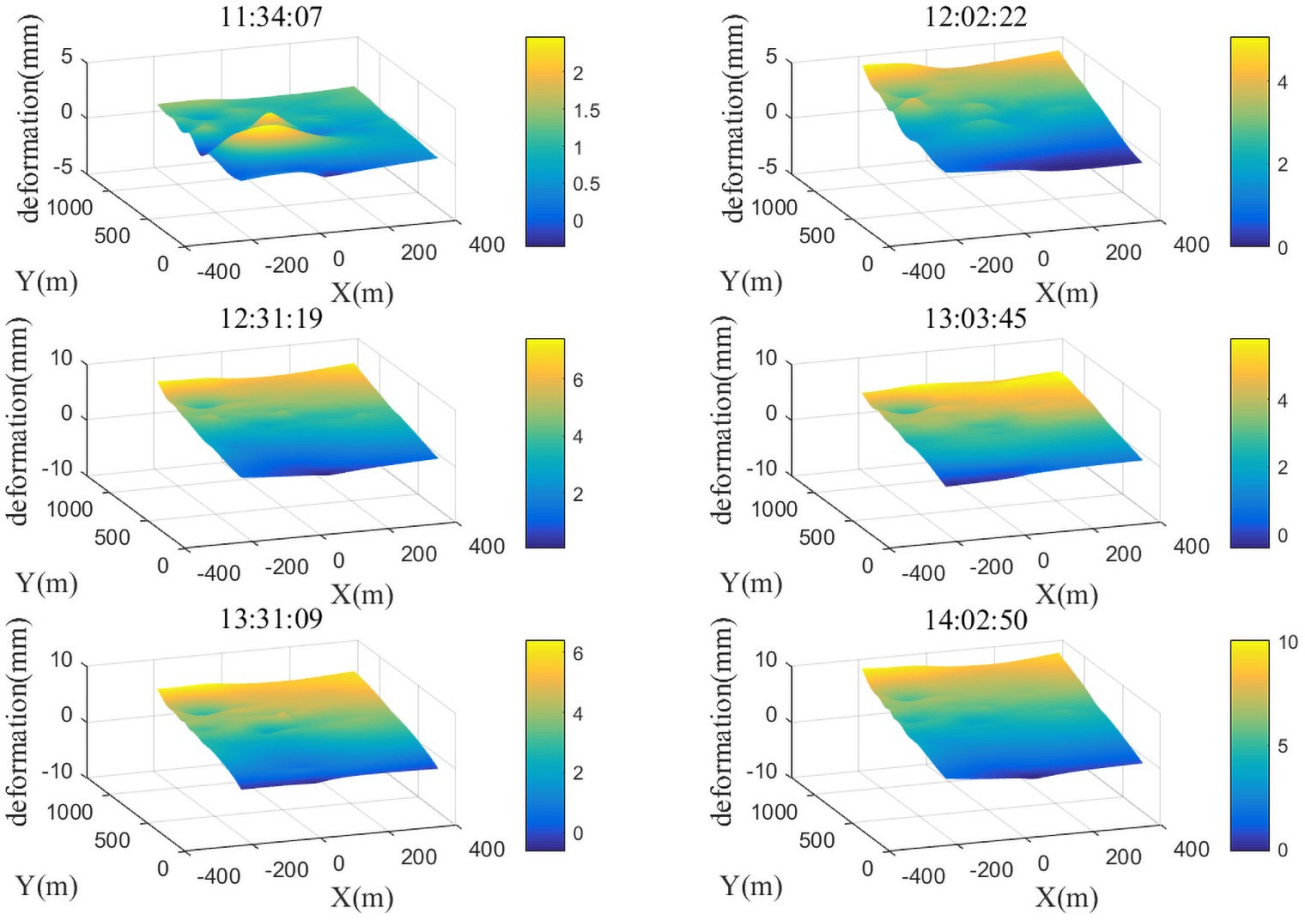
Atmospheric disturbance deformation map of target region at different time periods.

**Fig 5:**
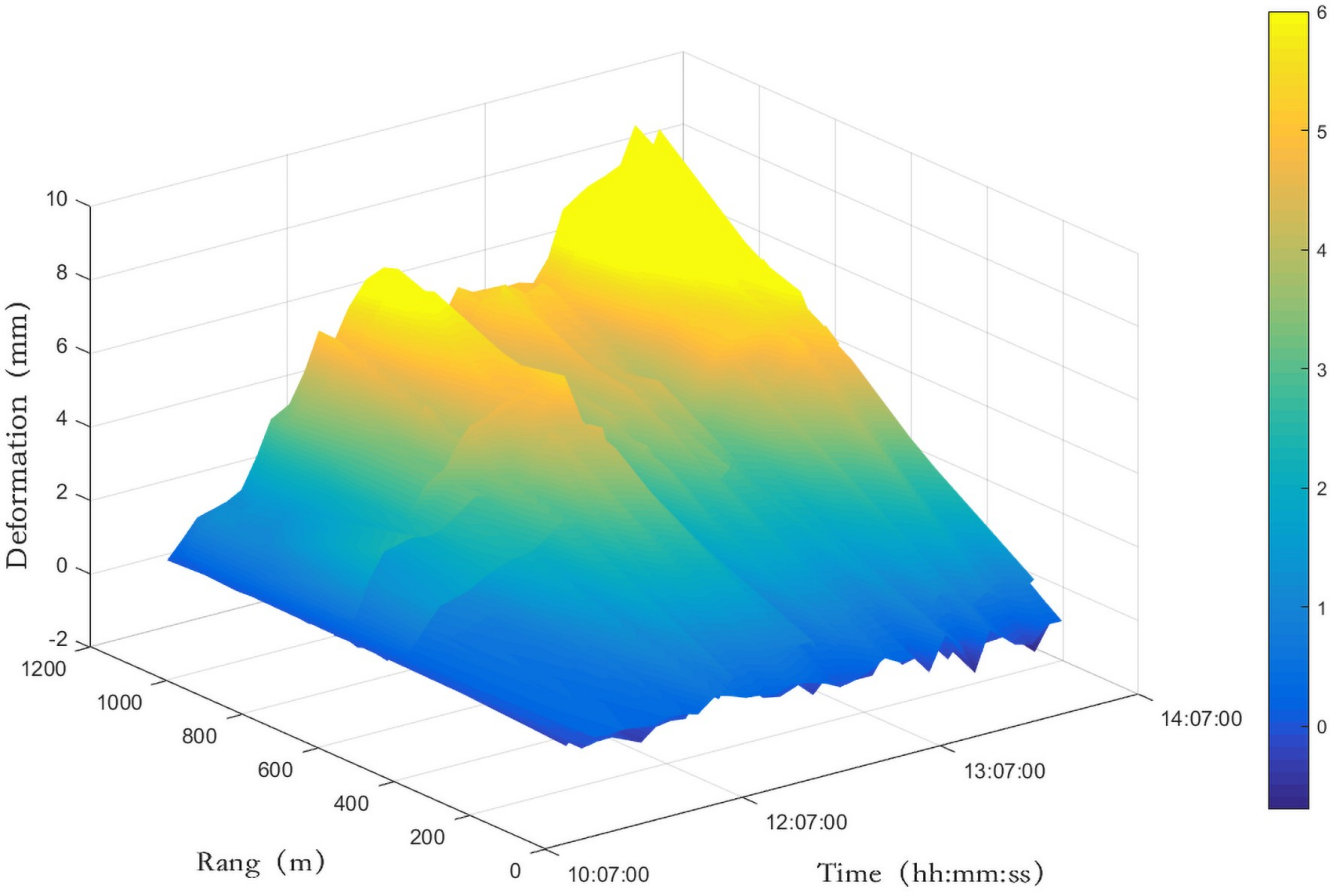
Atmospheric disturbance deformation map at different stadia.

With the first radar image acquired during the observation period as the benchmark, namely *n*(1) = 0, six radar images for the following observation periods were selected: 11:34:07, 12:02:22, 12:31:19, 13:03:45, 13:31:09, and 14:02:50. The six radar images were processed for interference and the deformations caused by meteorological disturbance are shown in Figs 4 and 5. Fig 4 shows that, with the exception of the first observation period, the atmospheric disturbance effects of the other observation periods are quite consistent. The influence in the range direction (Y axis) shows a linear increment, with a cumulative deformation of 10 mm during the observation period. The influence in the azimuthal direction is relatively small, mostly less than 1 mm. In some areas, the effect can be as large as 2 mm, such as the region 600 m from the radar in the range direction in the first radar image and the region 900 m from the radar in the second image. Combined with on-site data, it was found that during the first observation period the power plant executed a draining operation and generated a large quantity of water vapor, causing the meteorological disturbance of the region to be different from that in other regions in this time period. The results in Fig 5 show that the atmospheric disturbance has greatly influenced the radar interferometric phase, causing a cumulative deformation of 10 mm within 3 h. The effects were the strongest near 12:00 and 14:00, the effect gradually increased from 10:00 to 12:00, decreased in the 12:00–13:00 time period, and then continued to increase during 13:00–14:00.

**Fig 6:**
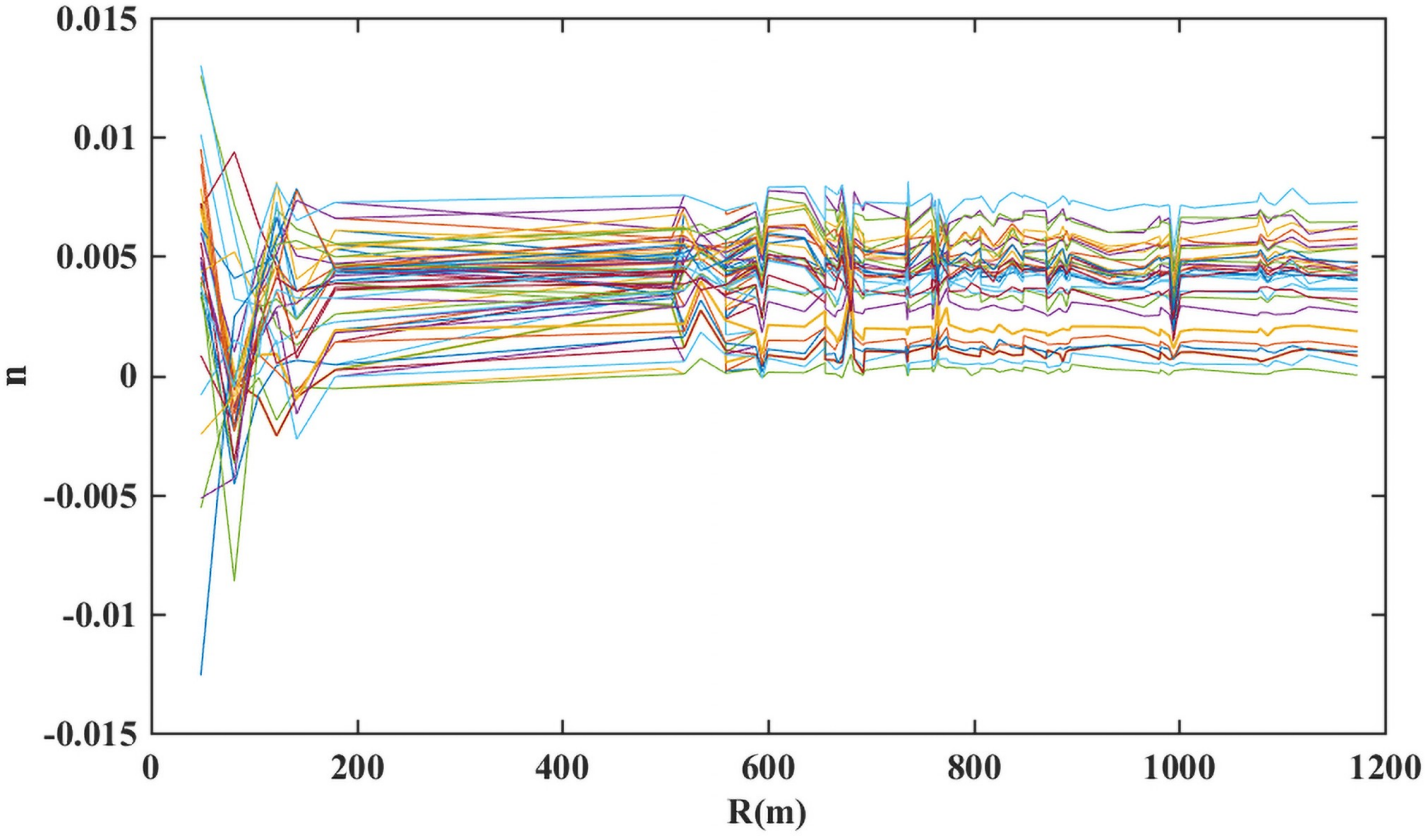
Atmospheric refraction coefficient at different distances.

**Fig 7:**
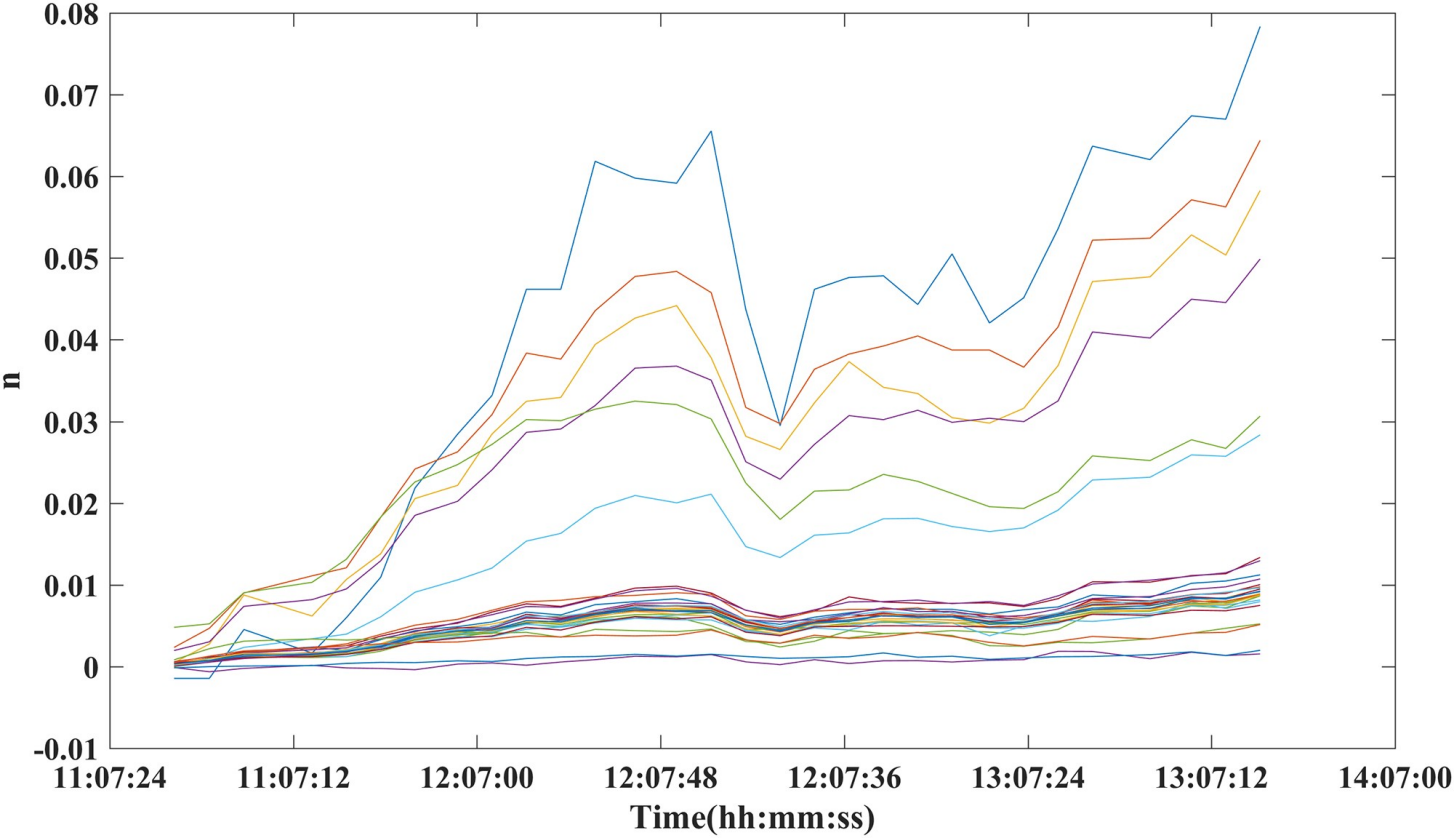
Atmospheric refraction coefficient at different time periods.

The atmospheric refraction index of the selected PS points in the observation period was calculated using Eq (6), and the distribution characteristics for different distance and at different observation times were statistically analyzed to study the effects of meteorological factors on the interferometric phase at different distances and times. The results are shown in Figs 6 and 7. In Fig 6, each characteristic line indicates the atmospheric refractive index at different distances from the same observation period PS in the same observation time period. In Fig 7, each characteristic line indicates the atmospheric refractive index obtained at the same PS point at different points in time. The results in Figs 6 and 7 show that the atmospheric refractive index is more consistent for stable points located more than 500 m from the radar in the line-of-sight direction. Within the same observation period, their atmospheric refractive index is basically the same. However, the trend of deformation change for stable points within 200 m of the radar is distinctly different from those of other stable points; their range of change of the refractive index is considerably greater than that of other stable points. This indicates that there are definite differences in atmospheric refractive index in different regions of the observation zone.

**Fig 8:**
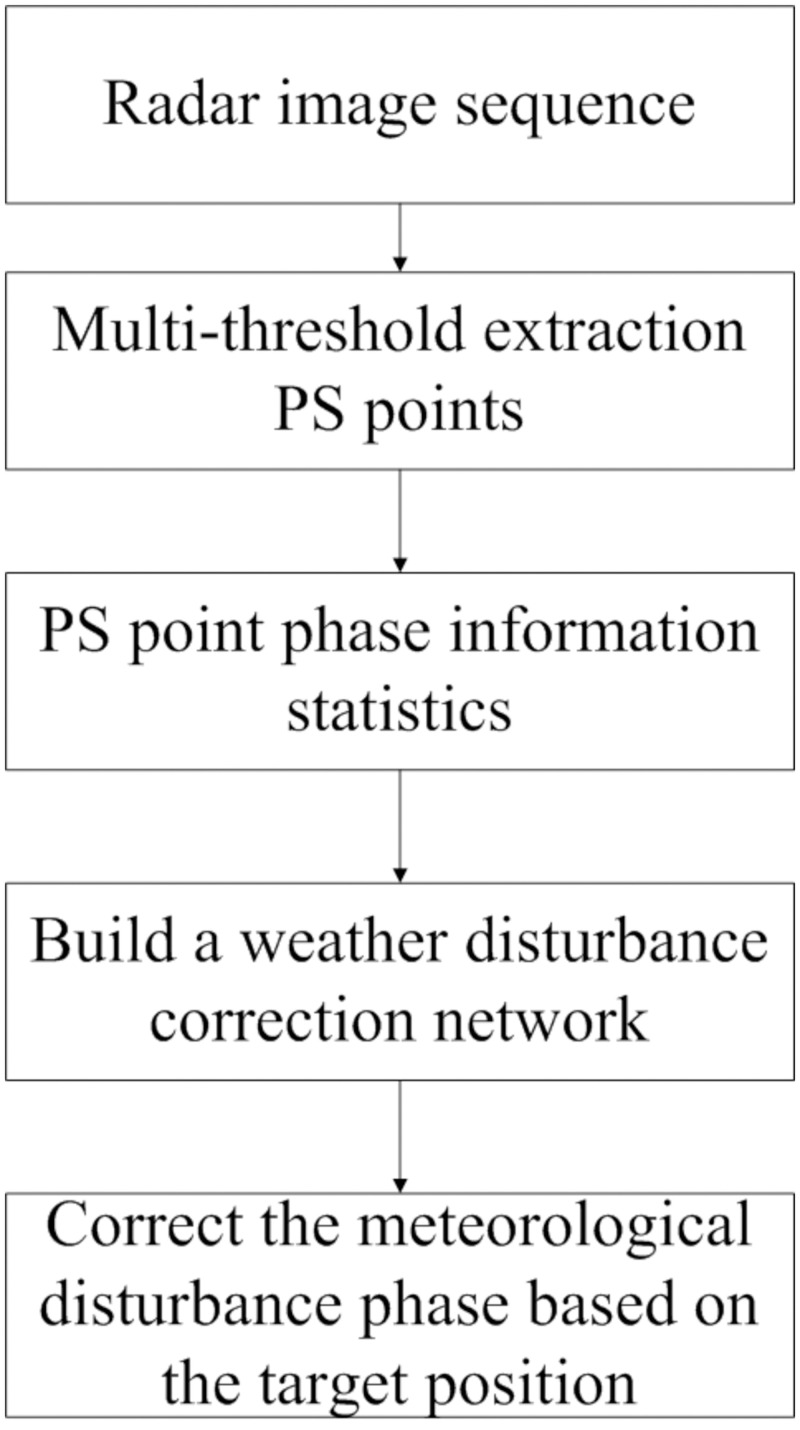
Flowchart of atmospheric correction based on target location information.

Considering the distribution characteristics of the atmospheric refractive index in the range and azimuthal directions, there should be a distance threshold when making atmospheric corrections to eliminate stable points with an atmospheric refractive index that is different from that in the target zone. In addition, for line-of-sight distances 1,000 m or greater, the azimuthal effect of meteorological factors should also be considered. In this paper, a correction method for meteorological disturbances that takes into account the target position information is proposed. First, the PS points are chosen within the observation scenario using multiple thresholds. A threshold is established according to distribution characteristics of the atmospheric refractive index in the range direction, and the phase of the chosen points is analyzed statistically. Stable points within the observation period are chosen and a meteorological correction network is established. Finally, the correction network is combined with the positions of the target points to choose two points that are the closest to the target in both the range and azimuthal directions. Based on the two chosen points, the meteorological disturbance phase of the target point is calculated by interpolation and corrected. The data processing flowchart is shown in Fig 8.

## Deformation analysis of feature points of plant building and dam

**Fig 9:**
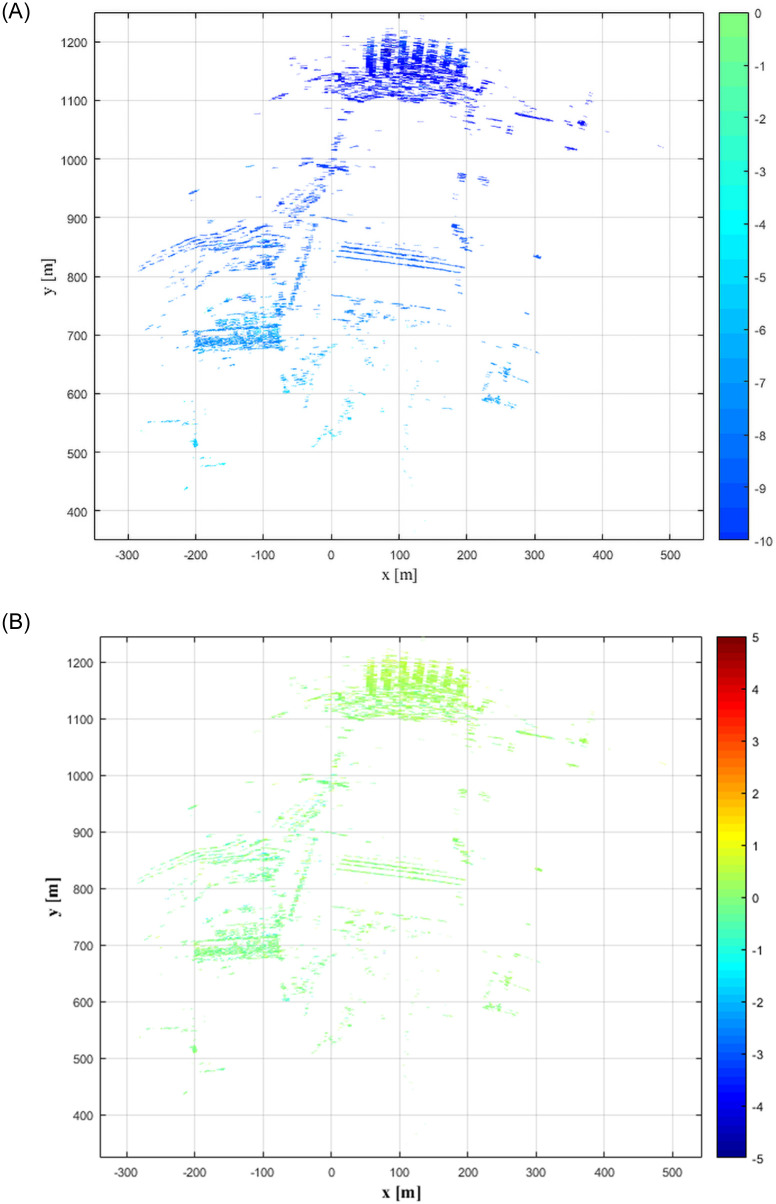
Cumulative deformation of target points during observation period: (A) Uncorrected and (B) corrected results. A negative value indicates that the deformation is toward the line of sight of the radar, and a positive value indicates that the target is deformed away from the line of sight of the radar.

Ten stable target points of the power plant and dam acquired during the observation period were chosen as feature points: P1–P5 were selected as feature points of the power plant, and P6–P10 feature points of the dam. These points were known to have not moved during the observation period through *a* priori information, so the measured deformation of these points may be regarded as errors caused by the atmospheric disturbances. Fig 9 shows the target deformation error before and after correction. The figure shows that the cumulative atmospheric effects on the target points during the observation period are 4–11 mm, and the body of the dam, located farthest from the radar, experienced the greatest effect, exceeding 9 mm. After meteorological correction, the effects of the atmosphere on the target points are less than 1 mm and the observation results accurately reflect the true deformation of the targets. Therefore, in the observation of target motion with radar interference, it is necessary to suppress the effects of meteorological disturbances.

**Fig 10:**
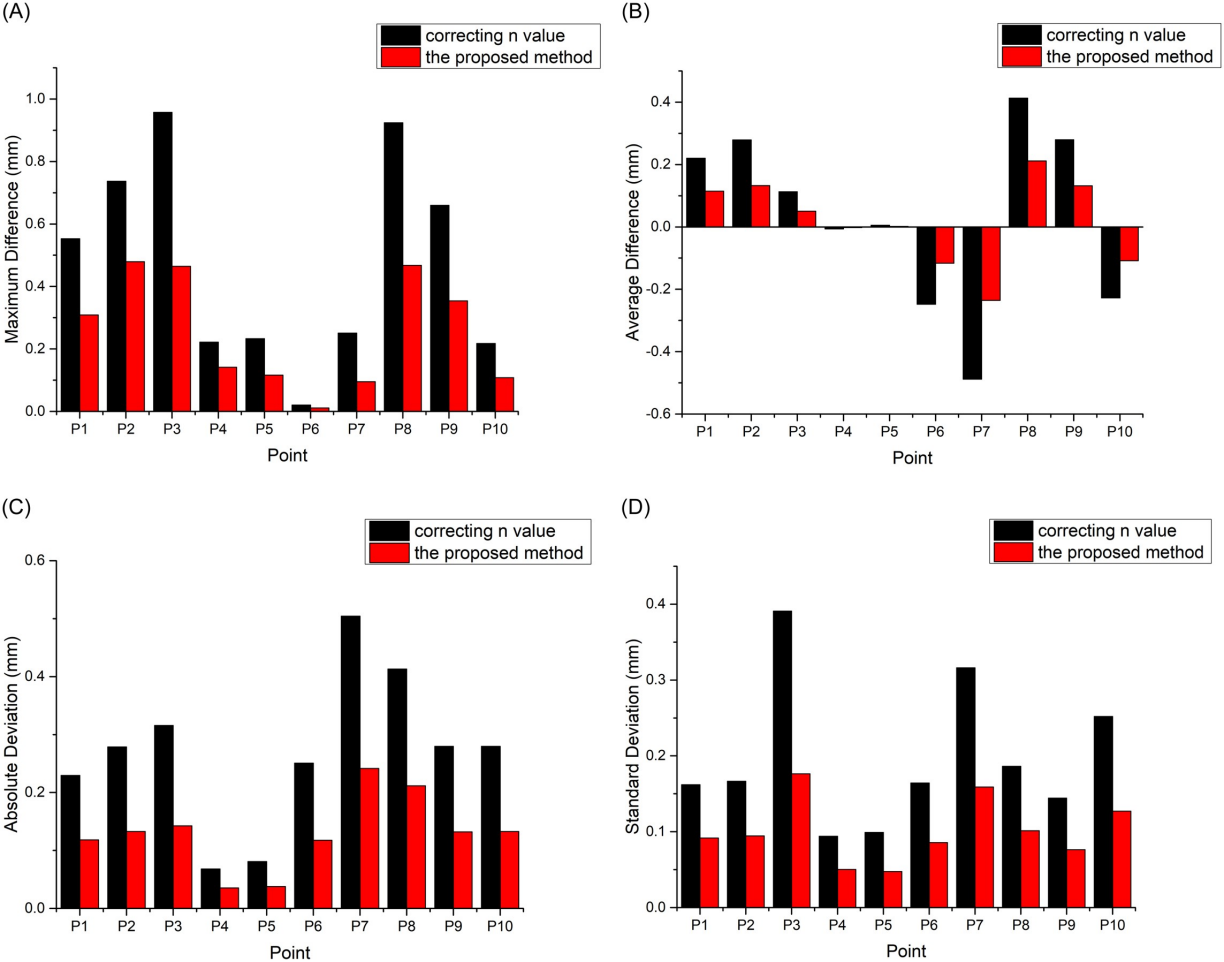
Bar charts of meteorological corrections at feature points:(A) Maximum difference, (B) mean difference, (C) absolute difference, and (D) standard deviation.

Meteorological corrections were separately performed on the feature points using the n-value correction method described previously [5] and the method proposed in this paper. Fig 10 shows a comparison of the maximum difference, mean difference, absolute difference, and standard deviation of the meteorological disturbances at each point during the observation. The comparison results show that feature point P6 has the least meteorological disturbance phase at each time (less than 0.1 mm after correction), but the meteorological effect at this point decreases linearly on the timescale, resulting in other effect values at this point not being the smallest. The mean value of the meteorological effect over the entire observation cycle is almost zero at points P4 and P5, with an absolute difference value less than 0.1 mm and a small standard deviation, but there are still some 0.3-mm effects at certain times. The corrections are not so effective for points P3 and P8, with the maximum difference still near 1 mm. There are therefore feature points with good correction results and points with poor correction results at different ranges, leading to spatial uncertainties in the meteorological correction effects.

According to analysis of the four indicators of corrections, the method proposed here is superior to the method of Ref. [5] in all aspects. Here, the maximum difference of the feature points decreased on average by 47%, from 0.477 to 0.255 mm. The mean difference decreased by 55% on average, from 0.034 to 0.018 mm. The absolute difference value decreased on average by 51%, from 0.27 to 0.13 mm. The standard deviation decreased on average from 0.198 to 0.1 mm. In addition, of all the feature points, point P3 has the best correction results in maximum difference, absolute difference, and standard deviation value. The maximum difference decreased by 52%, from 0.96 to 0.46 mm; the absolute difference by 56%, from 0.32 to 0.14 mm; and the standard deviation by 54%, from 0.39 to 0.18 mm. Point P5 has the best correction results in mean difference, decreasing by 68%, from 0.0055 to 0.0017 mm.

## Conclusions

Ground-based radar interferometry is greatly affected by atmospheric factors in the observation environment, especially the moisture content of the atmosphere. When a large amount of water vapor is present in the radar observation environment, the effects of meteorological disturbance are different in the range direction and azimuthal direction.

The phase of meteorological disturbance increases linearly in the range direction and has a relatively large effect on the measurement value. If the phase is left uncorrected, it can quickly turn into a 10-mm false displacement and jeopardize the accurate analysis of the target. The phase effect in the range direction can be effectively weakened with the distance polynomial using known stable points in the observation scene as a priori information.

The phase effect of meteorological disturbance in the azimuth direction is random and exhibits no evident linear relationship. Its effect on the observation value is smaller than that in the range direction. For observation scenes that are complex, such as those that contain abundant water vapor, the effect in the azimuthal direction can reach 2 mm and reduce the observation accuracy; it must therefore be corrected effectively.

The correction method for meteorological disturbance based on location information can produce good correction results. First, PS points are selected in the observation scene, and these points are then used to calculate the phase distribution characteristics in the observation scene and construct the meteorological disturbance correction model for different observation times. A regional interpolation method is used to correct the phase of the target. This method is better than the distance polynomial method and lowers the maximum difference, mean difference, absolute difference, and standard deviation of meteorological effect by averages of 47%, 55%, 51%, and 48% respectively, thus effectively improving the accuracy of radar observation.
